# Aural Complaints in Consequence of Malaria

**Published:** 1880-11

**Authors:** F. C. Hotz

**Affiliations:** Chicago


### Notes from Private Practice.

### Article V.

Aural Complaints in Consequence of Malaria. A Case of Primary Post Aural Abscess without Implication of the Middle Ear. *Archives of Otology*, Vol. IX, No. 3, Sept. 1, 1880. By F. C. Hotz, m.d., Chicago.

Under the first of the above heads appears a brief summary of the literature of malaria in its relation to affections of the ear, supplemented by the narration of three well marked cases which had been patiently and persistently treated by topical measures without effect, but yielded readily and permanently to the administration of quinine. The first and third cases are so interesting that we deem a summary of them to be of great importance to the general practitioner. For the same reason we give also Dr. Hotz' general conclusions based upon those cases.

Lady, thirty-five years. Subject to chronic nasal catarrh and sore throat. March 1876, acute aggravation. Throat symptoms relieved by nitrate of silver. On the advice of some "good friend," cold water was drawn into the nose. Sharp and severe pains and fullness was immediately felt in right ear. Agonizing pains during night. Ear felt as if filled with water. Watch not heard at all. Shrapnell's membrane being distended, was punctured; leeches applied, but relief was only temporary.

April 4th, the day following, external meatus filled with pus; pain moderate in morning, became severe at night. Mastoid process was tender but devoid of redness or swelling. External meatus was so sensitive that the mere gentle injection of tepid water was exceedingly painful. Leeches, poultices, turpentine emulsion produced no benefit.

April 7th, four grains of quinine every two hours. Free from pain all night; no tenderness over mastoid process; injection of water gave no pain. April 15th, discharge ceased and perforation healed. Patient still complained of a little fullness, which was relieved by the air douche, and a continuation of small doses of quinine.

On the above case Dr. Hotz remarks: The inflammatory process so clearly started by the cold water could not be reduced by local treatment. Paracentesis of the drum-head added to the severity of inflammation. A remarkable disproportion existed between the subjective and objective symptoms.

The third case, male forty-eight years. Very annoying soreness about the left ear and left side of head, particularly in region of left parietal bone. Slight redness over mastoid region, no tenderness or swelling. Redness probably due to irritants used. Two swollen and tender glands in sub-auricular fossa. External meatus red and swollen so that inspection of membrana tympani was difficult. The membrane, however, was perforated, and the edges red and swollen. It had been of three weeks' standing, and the patient, according to his family physician, had had several attacks of syphilitic affections of the bones. Watch was not heard at all by left ear.

The result of treatment, extending over four weeks, Dr. Hotz sums up as follows: Constitutional treatment by iodide of potassium, ten grains three times a day, had accomplished nothing, and local remedies had shown a decidedly bad effect; the present state of ear about the same as before treatment.

Questioning the patient again for more light on the history of the case, Dr. Weber Liel's *otitis intermittens* revealed itself, with the general characteristic of malaria. April 8th, began taking twenty grains of quinine daily, and from that time the disorder began to disappear; and on April 20th head was free from pain, meatus of normal width, no discharge from ear, opening in drum-head closed; can hear the voice very well, and watch, when in contact.

The intolerance of the ear for local remedies together with the disproportion between the morbid changes in the ear and the degree and extent of the nervous symptoms, may be regarded as good evidence of the malarial character of the aural affection.

Under the second heading the following case, here briefly summarized, is detailed and discussed:

Carpenter, forty-five years. No previous history of ear trouble except defective hearing. Awoke in the night of January 15, 1878, with violent pain behind left ear; in the morning the left mastoid was swollen and tender. Blisters, leeches, poultices, produced no benefit, but relief from pain occurred with the appearance of pus in the external auditory canal. After two weeks the discharge stopped and pain returned; this alternation continued for six months, during which time the swelling gradually extended down the tissues of the neck. General health gave way, appetite and sleep forsook him, and his brain became so irritable that he could not read two lines without feeling dizzy and nauseated.

On July 24th, when Dr. Hotz saw him for the first time he was pale, emaciated, despondent. The integument of the left auricle and post aural region was deep red. Considerable swelling of post aural region, no tenderness. Swelling slight over mastoid region, but increased at the top of mastoid process, marked and fluctuating in sub-auricular fossa; upper end of sterno-cleido-mastoid muscle swollen, and head kept inclined to left shoulder, with face turned to right side. Change from this position and opening of the mouth was painful.

External auditory canal was neither red nor swollen, no perforation of membrane was found, but in the posterior wall at the juncture of the cartilaginous portions, was a small elevation with a minute opening at its vertex. A small probe introduced into this opening could not be advanced towards the middle ear or the mastoid cells, but passed readily toward the top of mastoid process; and on pressure in the sub-auricular fossa muco-purulent matter exuded. The hearing in both ears was almost equally defective.

The limited extent of the affection after so long a period—six months—the swelling of the upper end of sterno-cleido-mastoid muscle, the fistulous opening at the juncture of the cartilaginous with the bony part of external meatus with the fact that a probe could be passed in the direction of the mastoid process determined the diagnosis as subperiosteal abscess.

A deep incision, July 27, was made behind the ear from the line of the orifice of the external meatus to the insertion of sterno-cleido-mastoid muscle. No pus; periosteum and bone healthy, but on extending the incision one inch further down into the more swollen tissues of the sub-auricular region, a cavity was reached containing a large amount of muco-purulent secretion. This cavity was demonstrated to connect with the opening into the external meatus. The wound being cleaned and a drainage tube kept in it, all distressing symptoms soon disappeared. On September 8th, wound behind the ear was cicatrized. External auditory canal as normal as the other side.

